# Development of three ecological spectroscopic methods for analysis of betrixaban either alone or in mixture with lercanidipine: greenness assessment

**DOI:** 10.1098/rsos.211457

**Published:** 2022-02-02

**Authors:** Amal A. El-Masry, Dalia R. El-Wasseef, Manal Eid, Ihsan A. Shehata, Abdallah M. Zeid

**Affiliations:** ^1^ Department of Medicinal Chemistry, Faculty of Pharmacy, Mansoura University, 35516 Mansoura, Egypt; ^2^ Department of Pharmaceutical Analytical Chemistry, Faculty of Pharmacy, Mansoura University, 35516 Mansoura, Egypt; ^3^ Department of Pharmaceutical Chemistry, Faculty of Pharmacy, Delta University for Science and Technology, 35712, Gamasa, Egypt

**Keywords:** betrixaban, lercanidipine, difference spectrophotometry, derivative spectrophotometry, greenness evaluation, dosage forms

## Abstract

Three eco-friendly spectrophotometric methods were developed for determination of the novel anticoagulant drug, betrixaban (BTX). The first method (method A) was based on direct analysis of BTX at 229.4 nm on the zero-order spectrum using methanol as the optimum solvent. While the second method (method B) was based on measuring difference absorption value (ΔA) of BTX at 335 nm, which was obtained from pH-induced spectral difference (difference spectra of BTX in 0.1 M NaOH versus 0.1 M HCl). The third method (method C) was based on measurement of the first-derivative amplitudes of BTX and its co-administered Ca channel blocker lercanidipine (LER) at 304 and 229 nm for simultaneous assay of BTX and LER, respectively. All methods were linear over concentration ranges of 1.0–20.0 and 8.0–80.0 µg ml^−1^ for BTX in methods A and B, respectively, and of 1.0–20.0 and 1.0–25.0 µg ml^−1^ for BTX and LER, respectively, in method C. The three methods were fully validated and assessed for greenness by three metrics: analytical eco-scale, green analytical procedure index and Analytical GREEnness metrics. The results indicated the validity and greenness of the proposed methods. Moreover, the methods were applied to assay the studied analytes in their dosage forms with high percentage of recovery and low percentage of relative s.d. values.

## 1. Introduction

**Figure 1.**
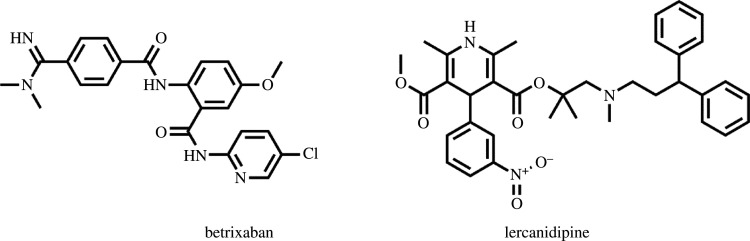
Chemical structures of betrixaban and lercanidipine.

The novel class, non-vitamin K oral anticoagulants provides better therapeutic control with low bleeding complications when compared with other anticoagulant agents [1]. Betrixaban (BTX) is a novel synthetic, potent and selective oral anti-coagulant drug. Its chemical name is N-(5-chloropyridin-2-yl)-2-([4-(N,N-dimethylcarbamimidoyl)benzoyl]amino)-5-methoxybenzamide [2] (figure 1). BTX is mainly used in treatment of pulmonary embolism and venous thromboembolism by direct reversible competitive inhibition of Xa factor. Co-administration of BTX with other Ca channel blocker was very effective in management of atrial fibrillation [3], where the normal blood flow can be interrupted with the irregular heart beat leading to potential risks of blood clots and strokes. Therefore, BTX as an anti-coagulant will prevent formation of blood clots, while a Ca-channel blocker such as lercanidipine (LER) will relax the arterial muscles and reduce the overall heart rate. LER is chemically named as (±)-2-[(3,3-diphenylpropyl)methylamino]-1,1-dimethylethylmethyl1,4-dihydro-2,6-dimethyl-4-(*m*-nitrophenyl)-3,5pyridinedicarboxylate [4] (figure 1).

To the best of our knowledge, there are four published high-performance liquid chromatography (HPLC) methods for quantitative determination of BTX [5–8]. These four methods involved using tedious procedures and dealing with hazardous chemicals and solvents. Moreover, expensive instrumentation with high energy consumption and complicated set-up were used, which reduces the usability in quality control laboratories. On the other hand, there are several reported analytical methods for the assay of LER including: spectrophotometry [9–15], thin-layer chromatography (TLC) [16,17], HPLC [5,18–24], high-performance thin-layer chromatography (HPTLC) [17], ultra-performance liquid chromatography–tandem mass spectrometry (UPLC-MS/MS) [25,26], liquid chromatography–tandem mass spectrometry (LC-MS/MS) [21,27–30] and capillary electrophoresis [31].

Only one method was reported for simultaneous analysis of BTX and LER [5]. The reported method was based on HPLC assay of the studied analytes in different matrices. Although the method was very selective and precise, the use of expensive instrumentation, consumption of large volume of hazardous organic solvents and consumption of long time in analysis and preparation of the samples were the main drawbacks. Therefore, the use of a simple, timesaving, cost-effective, less hazardous and rapid spectrophotometric method for the simultaneous estimation of BTX and LER is highly advantageous.

To our knowledge, no spectrophotometric methods were developed for determination of the newly Food and Drug Administration (FDA) approved drug (BTX). Herein, this study aimed to develop new, simple, rapid and eco-friendly spectroscopic methods for quantification of BTX alone or with its co-administered drug (LER) in bulk and in laboratory-prepared combined pharmaceutical preparations.

## 2. Experimental

### 2.1. Instruments

Spectrometric analyses and investigations were performed using a Shimadzu UV-1601PC (Kyoto, Japan) UV-Vis double-beam spectrophotometer with a 1 cm path length quartz matched cuvette. Fast scan speed was adjusted in all recorded absorption spectra. Measurement of pH values was carried out using a Consort P 901 pH-meter.

### 2.2. Materials and reagents

BTX (99.8% labelled purity) was procured from Portola Pharmaceutical Company (China). LER of 99.5% labelled purity was obtained from Recordati Industria Chimica e Farmaceutica S.P.A. (Italy). Care_-_dipine tablets (purchased from local pharmacy), categorized to contain 10 mg LER, is a product of Pharmacare Egypt for Trading Agency. Methanol (HPLC high grade), magnesium stearate and dextrose were procured from Sigma-Aldrich (Germany). Both hydrochloric acid and sodium hydroxide were bought from El-Nasr Pharmaceutical Chemicals Co., Egypt.

### 2.3. General procedures

#### 2.3.1. Standard solutions

Methanolic standard solutions of BTX (1.0 mg ml^−1^) and LER (0.1 mg ml^−1^) were prepared by accurately transferring 0.1 and 0.01 g of BTX and LER, respectively, in two separate 100.0 ml volumetric flasks. Further dilution of BTX was performed to obtain working solution of 100 µg ml^−1^.

#### 2.3.2. Construction of calibration curves

##### 2.3.2.1. Direct determination of BTX by UV absorption spectroscopy (method A)

Accurately measured aliquots of working solution of BTX (100 µg ml^−1^) covering the final concentration range of 1.0–20.0 µg ml^−1^ were placed into a series of 10.0 ml measuring flasks. These flasks were filled with methanol to 10.0 ml. The zero-order absorption spectra of BTX were recorded against blank (methanol) at 229.4 nm at which maximum absorbance values of BTX were obtained. These absorbance values were then plotted against the final concentrations to get the calibration graphs.

##### 2.3.2.2. Determination of BTX by difference spectrophotometry (method B)

Working solutions of BTX (8.0–80.0 µg ml^−1^) were prepared by placing appropriate volumes of the stock solution (1.0 mg ml^−1^) in two series of 10.0 ml measuring flasks. Then, the volumes in the first and second series were adjusted with 0.1 M sodium hydroxide (NaOH) and 0.1 M hydrochloric acid (HCl), respectively, to get series of equimolar concentration of BTX. The UV absorption spectra were recorded immediately against blank solutions (0.1 M NaOH for the first series and 0.1 M HCl for the second series). The difference absorption spectra (ΔA spectra) were gathered by subtracting the absorption spectra of BTX in the second series (in 0.1 M HCl) from the corresponding spectra of BTX in the first series (in 0.1 M NaOH). Standard calibration curve was derived by construction of the relationship between ΔA values at 335 nm versus final BTX concentrations, followed by calculation of the corresponding regression equations.

##### 2.3.2.3. Simultaneous estimation of BTX and LER by first-order derivative spectrophotometry (method C)

In two separate sets of 10.0 ml measuring flasks, increasing volumes of the standard solution of BTX and LER (100 µg ml^−1^ each) were quantitatively transferred to give solutions within the concentration ranges of 1.0–20.0 µg ml^−1^ and 1.0–25.0 µg ml^−1^ for BTX (first set) and LER (second set), respectively. Then, the solution was completed with methanol to 10.0 ml. The zero-order absorption spectra for each drug individually were recorded against blank (methanol), followed by deriving the first-order derivatives. The absolute values of the first-order derivatives were measured at 304 and 229 nm for BTX and LER, respectively (zero crossing point of LER and BTX, respectively). Standard calibration curves were derived by constructing the relationship between the derivative amplitudes versus final concentrations, followed by calculation of the corresponding regression equations.

##### 2.3.2.4. Procedure for simultaneous estimation of BTX and LER in their laboratory-prepared mixture

Into series of 10.0 ml measuring flasks, aliquots (accurately measured) of the standard solutions of BTX and LER (100 µg ml^−1^ for both) were placed. Therefore, different laboratory-prepared mixtures with different concentrations were prepared in constant ratio of 4 : 1 for BTX : LER. After that, the subsequent steps operated under §2.3.2.3 were followed. Finally, the percentage recoveries were statistically calculated by using the corresponding regression equations.

##### 2.3.2.5. Procedures for the estimation of BTX and LER in their dosage forms

##### 2.3.2.5.1. Methods A and B

Ten laboratory-prepared BTX capsules (containing dextrose and magnesium stearate additives) were accurately weighed, ground and thoroughly blended for the assay of BTX content. An accurate weight equivalent to 0.1 g of BTX was transferred to 100 ml volumetric flask, diluted with methanol and sonicated for 10 min to ensure the complete solubility of all contents. The final prepared BTX stock solution (1000 µg ml^−1^) was furthermore diluted with methanol to obtain a 100 µg ml^−1^ working solution. Finally, the same steps described under §§2.3.2.1 and 2.3.2.2 for methods A and B were followed, and the nominal contents of BTX were determined from the corresponding derived regression equations.

##### 2.3.2.5.2. Method C

Ten laboratory-prepared BTX capsules were weighed, ground and blended for BTX analysis. Subsequently, 10 Care_-_dipine tablets were weighed, ground and thoroughly mixed to assay LER. An accurate weight equivalent to 0.1 g of BTX and 0.02 g of LER were transferred into two separate 100 ml measuring flasks, diluted with methanol and sonicated for 20 min. Further dilution using methanol was performed for BTX only to obtain working solution with concentration of 800 µg ml^−1^. Finally, the same steps described under §2.3.2.3 for method C were followed, and the nominal content of the studied drugs and the percentage recoveries were calculated using the corresponding regression equations.

## 3. Results and discussion

### 3.1. Optimization of the proposed spectrophotometric methods

**Figure 2.**
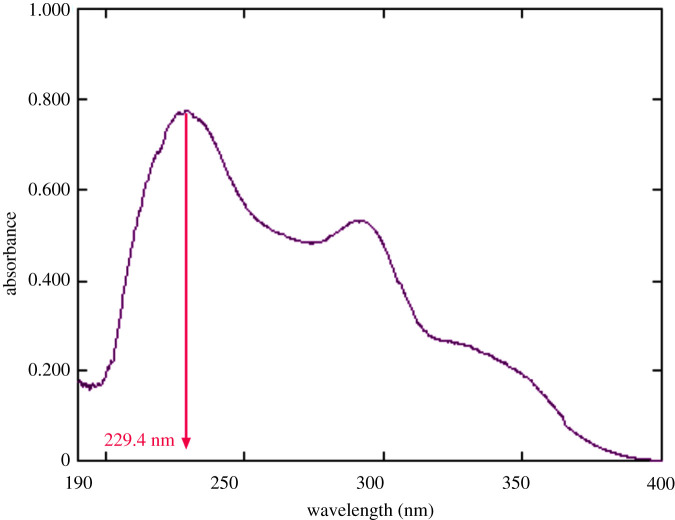
Absorption spectrum of BTX (15.0 µg ml^−1^) in methanol at 229.4 nm.

**Figure 3.**
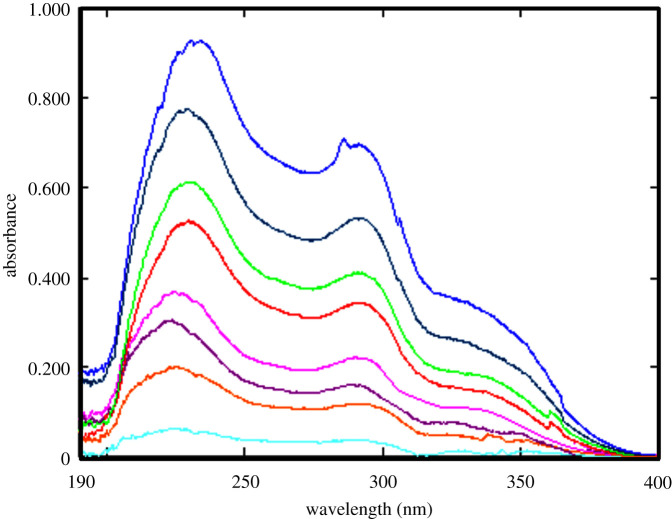
Absorption spectra of BTX (1.0, 3.0, 5.0, 7.0, 10.0, 12.0, 15.0, 20.0 µg ml^−1^) in methanol at 229.4 nm.

**Figure 4.**
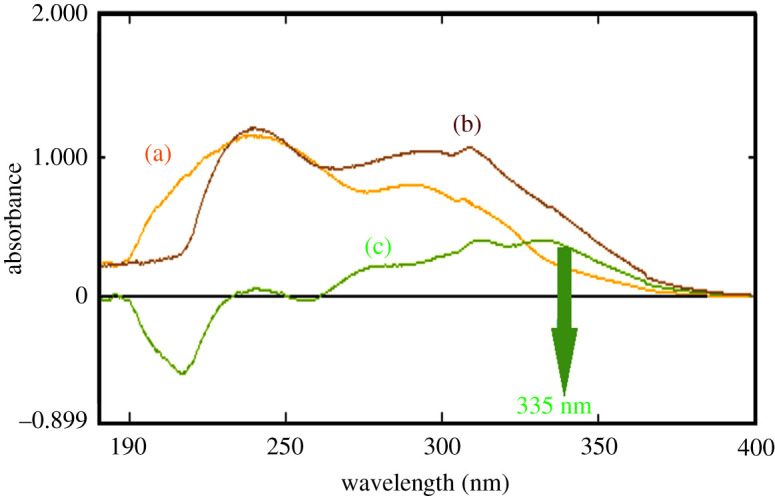
The UV absorption spectra of (a) BTX (30 µg ml^−1^) in 0.1 M HCl (yellow colour), (b) BTX (30 µg ml^−1^) in 0.1 M NaOH and (c) with BTX (30 µg ml^−1^) in 0.1 M NaOH versus BTX (30 µg ml^−1^) in 0.1 M HCl (ΔA spectrum).

The proposed spectrophotometric methods were optimized for the analysis of the novel anticoagulant drug, BTX alone or with its co-administered drug, LER. The first two methods (methods A and B) were based on individual determination of BTX in both pure and prepared dosage form depending on two different techniques: zero-order spectrophotometric method (method A) and eco-friendly difference spectrophotometric technique (method B). The UV absorption spectrum of BTX in methanol exhibited two maxima at 229.4 and 290.0 nm, as shown in figure 2. In method A, the direct quantitative determination of BTX was performed at 229.4 nm, as it possessed more linear and optimum accurate measurements, as shown in figure 3. In method B, the spectral difference between two equimolar basic and acidic solutions (0.1 M NaOH and 0.1 M HCl) of BTX is recorded. The two absorption spectra of BTX in acidic and basic equimolar solutions were drawn as shown in figure 4. Upon graphical analysis, it was found that bathochromic shift with hyperchromic effect was achieved in spectrum in basic solution when compared with equimolar acidic solution. This could be related to the basicity of BTX as its pka values were 10.91 and 11.61 [32]. In acidic medium, the nitrogen atom in BTX was protonated; therefore, neither conjugation could be occurred nor availability of lone pair. Whereas, alkaline medium possessed more availability of lone pair, so more conjugation could be achieved. Subtraction of the absorption spectra of BTX in 0.1 M HCl solution from the absorption spectra of BTX in 0.1 M sodium hydroxide was done, and the difference absorption spectra of BTX were constructed as demonstrated in figure 4. Isosbestic point is the point where it possesses zero difference absorbance value (ΔA). At this point, the same absorptivity must be achieved in both equimolar basic and acidic solution. Difference absorbance spectrum of BTX showed two isosbestic points at 238 and at 260 nm. The difference absorbance (ΔA) values were negative before wavelength of 238 nm, because the UV absorption of BTX in acidic medium was more than that achieved in equimolar basic solution. On the contrary, positive values of difference absorbance spectrum of BTX were obtained after wavelength 260 nm, thus because the UV absorption of BTX in acidic medium was less than that achieved in equimolar basic solution. Quantitative determination of BTX was measured at 335 nm, which showed maximum and accurate absorbance measurements.

**Figure 5.**
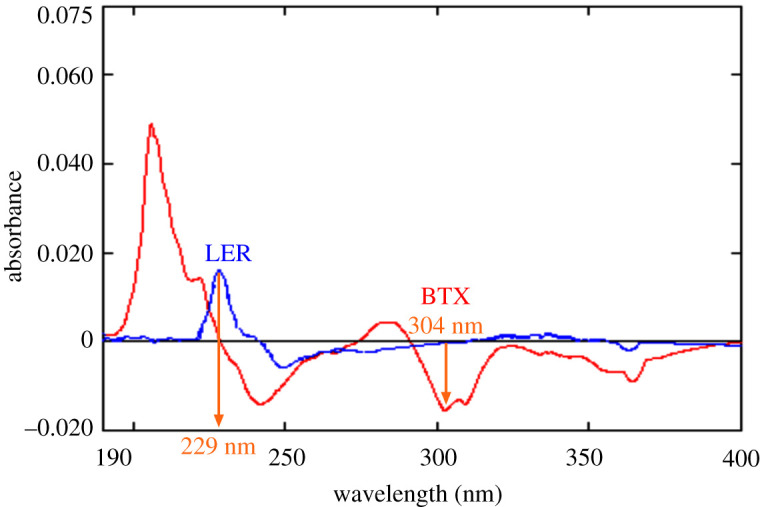
First-order derivative spectra of BTX (16 µg ml^−1^) and LER (4 µg ml^−1^) in methanol, where BTX was measured at 304 nm (zero crossing for LER), while LER was measured at 229 nm (zero crossing for BTX).

Method C was based on simultaneous determination of BTX with co-administered LER in prepared mixture and laboratory-prepared dosage form by application of first-order derivative technique. Because of difficulty in simultaneous determination of both drugs by the conventional direct spectrophotometric determination, the first derivative was the second option, as the detectability of minor features in the UV absorption spectrum was enhanced. The first-derivative spectra of both drugs (BTX and LER) were drawn as shown in figure 5. Upon displaying spectrum features of both drugs, it was found that first-order amplitudes at 304 nm (zero crossing of LER) and 229 nm (zero crossing of BTX) were the best to be chosen in simultaneous determination of BTX and LER, respectively.

### 3.2. Validation of the proposed methods

Validations of the three spectrometric methods were performed according to ICH guidelines [33] with respect to linearity and range, detection and quantification limits, accuracy, intra-day and inter-day precision and specificity.

#### 3.2.1. Linearity and range

The linearity of the three methods was investigated by plotting absorbance (A), difference absorbance (ΔA) and first-derivative amplitude (D^1^) versus drug concentration in methods A, B and C, respectively. The ranges of linearity for BTX were found to be 1.0–20.0 µg ml^−1^ in methods A and C and 8.0–80.0 µg ml^−1^ in method B. On the other hand, the linearity range of LER was found to be 1.0–25.0 µg ml^−1^ in method C as abridged in table 1. Linear regression analyses of the obtained data gave the subsequent equations:

**Table:** 

A = 0.02 + 0.05 C (*r* = 0.9999)	for BTX at 229.4 nm (method A)	
ΔA = −0.007 + 0.01 C (*r* = 0.9999)	for BTX at 335 nm (method B)	
D^1^ = 0.0001 + 0.001 C (*r* = 0.9999)	for BTX at 304 nm	(method C)
D^1^ = −0.001 + 0.005 C (*r* = 0.9999)	for LER at 229 nm

**Table 1.** Analytical data for the assay of BTX and BTX/LER through the laboratory-performed different spectrophotometric methods.

methods	method A	method B	method C	
parameter	BTX		BTX	LER
	229.4 nm	335 nm	304 nm	229 nm
concentration range (µg ml^−1^)	1.0–20.0	8.0–80.0	1.0–20.0	1.0–25.0
correlation coefficient	0.999			
slope (b)	0.05	0.01	0.001	0.005
intercept	0.02	−0.007	0.0001	−0.001
LOD^a^ (µg ml^−1^)	0.15	0.91	0.23	0.23
LOQ^b^ (µg ml^−1^)	0.45	2.74	0.71	0.69
*S_y/x_*^c^	3 × 10^−3^	4 × 10^−3^	1 × 10^−4^	5 × 10^−4^
*S_a_*^d^	2 × 10^−3^	3 × 10^−3^	7 × 10^−5^	3 × 10^−4^
*S_b_*^e^	2 × 10^−4^	6 × 10^−5^	1 × 10^−5^	2 × 10^−5^
% RSD^f^	0.83	0.80	0.90	0.69
% Er^g^	0.29	0.30	0.34	0.26

^a^Detection limit.

^b^Quantitation limit.

^c^Standard deviation of residuals.

^d^Standard deviation of intercept.

^e^Standard deviation of slope.

^f^Percentage of relative standard deviation.

^g^Percentage of relative error.

Where A is the absorbance, ΔA is the difference absorbance, D^1^ is the first-derivative amplitude, C is the drug concentration (µg ml^−1^) and *r* is the correlation coefficient.

#### 3.2.2. Quantification and detection limits

Limit of quantification (LOQ) and limit of detection (LOD) reflect the lowest concentration that can be quantitatively measured or readily detected, respectively, by the proposed methods. These values were calculated (table 1) by these equations,

3.1 $$\mathrm{LOQ}=\frac{10S_{a}}{b}$$

and

3.2 $$\mathrm{LOD}=\frac{3.3S_{a}}{b},$$

where *S_a_* represents the s.d. of the intercept and *b* is the slope of regression line of the calibration graph.

#### 3.2.3. Accuracy

The accuracy was determined by applying the general procedures for determination of the studied drugs and comparing the percentage recovery of pure samples of intact drugs, laboratory-prepared mixtures and laboratory-prepared dosage forms with another comparison method [5] over three concentrations levels covering the linearity range of each drug in each method. Statistical analysis of the resulted data was performed using Student's *t*-test and variance ratio *F*-test [34]. The results indicated the accuracy of the performed methods, as no significant differences were achieved, as shown in tables 2 and 3.

**Table 2.** Analytical data for the assay of betrixaban (BTX) and lercanidipine (LER) in raw material by the investigated different spectrophotometric methods and the other comparison ones. The values between brackets are tabulated *t-* and *F*-values at *p* = 0.05.

parameter	proposed methods				comparison method [5]	
	method A	method B	method C			
drug	BTX		BTX	LER	BTX	LER
	229.4 nm	335 nm	304 nm	229 nm		
$\bar{X}$^a^ ± s.d.	99.85 ± 0.83	100.24 ± 0.8	99.42 ± 0 90	100.45 ± 0.69	99.69 ± 1.14	99.86 ± 1.03
*t-*value	0.28 (2.23)	0.95 (2.26)	0.43 (2.26)	0.99 (2.26)		
*F*-value	1.88 (4.35)	2.01 (4.76)	1.63 (4.76)	3.67 (4.76)		

^a^Each result is the mean recovery of three individual analyses.

**Table 3.** Comparative analytical results for individual assay of BTX in laboratory-prepared tablets and simultaneous assay of BTX/LER combination in their laboratory-prepared mixtures and dosage forms by the three spectrophotometric methods. The values between brackets are tabulated *t-* and *F*-values at *p* = 0.05.

	parameter	proposed method (A)	proposed method (B)	comparison method [5]	
prepared dosage form BTX with additive^a^	$\bar{X}\pm s.d.$	99.72 ± 0.69	100.32 ± 0.73	99.86 ± 0.6	
	*t-*value	0.23 (2.13)	0.87 (2.13)	—	
	*F*-value	1.35 (19)	1.47 (19)	—	

	method (C)				
		proposed method		comparison method [5]	
	parameter	BTX	LER	BTX	LER
BTX + LER (4: 1) synthetic mixture	$\bar{X}\pm s.d.$	100.26 ± 0.61	99.54 ± 0.88	100.03 ± 0.14	99.42 ± 0.77
	*t-*value	0.63 (2.13)	0.17 (2.13)	—	
	*F*-value	17.07 (19)	1.31 (19)	—	
prepared BTX with additive^a^ and Care_-_dipine^b^		BTX	LER	BTX	LER
	$\bar{X}\pm s.d.$	99.72 ± 0.81	99.58 ± 1.14	100.07 ± 0.71	100.17 ± 1.15
	*t-*value	0.056 (2.13)	0.63 (2.13)	—	
	*F*-value	1.23 (19)	1.02 (19)	—	

^a^BTX with additive: prepared by mixing BTX with dextrose and magnesium stearate.

^b^Care_-_dipine tablet (Each tablet contains 10 mg LER).

#### 3.2.4. Precision

The repeatability (intra-day precision) and intermediate precision (inter-day precision) of the developed methods were assessed by analysis of three concentrations of each analyte, three times a day and for three consecutive days, respectively, for the three proposed methods. The small values of percentage of relative s.d. (%RSD) (less than 1.5%) ensured the high precision of the proposed methods and the perfect reproducibility of the results, as shown in table 4.

**Table 4.** Precision data for analysis of BTX and LER by the three spectrophotometric methods.

methods		method A			method B		
drug		BTX Concentrations (μg ml^−1^)					
parameters		8.0	10.0	12.0	20.0	40.0	60.0
intra-day	$\bar{X}$^a^	99.16	99.53	100.26	99.71	99.80	100.41
	± s.d.	0.64	0.71	0.67	0.48	0.64	0.44
	% RSD	0.64	0.71	0.67	0.48	0.64	0.44
	% error	0.37	0.41	0.38	0.28	0.37	0.25
inter-day	$\bar{X}$^b^	99.11	99.88	100.05	100.22	99.78	101.05
	± s.d.	1.11	1.03	0.89	0.97	0.78	1.2
	% RSD	1.11	1.03	0.89	0.97	0.78	1.2
	% error	0.64	0.59	0.52	0.56	0.45	0.68

methods		method C					
drug		BTX concentrations (μg ml^−1^)			LER concentrations (μg ml^−1^)		
parameters		8.0	10.0	12.0	6.0	12.0	18.0
intra-day	$\bar{X}$^a^	99.43	98.67	100.44	99.35	100.56	100.78
	±s.d.	0.97	0.42	0.74	0.91	0.63	0.51
	% RSD	0.97	0.42	0.74	0.91	0.63	0.51
	% error	0.56	0.24	0.43	0.53	0.36	0.29
inter-day	$\bar{X}$^b^	98.92	99.22	100.25	99.56	100.44	99.87
	±s.d.	1.10	0.85	0.68	1.02	0.82	0.69
	% RSD	1.10	0.85	0.68	1.02	0.82	0.69
	% error	0.64	0.49	0.39	0.59	0.47	0.4

$\bar{X}$^a^: Mean recovery of triple analyses of the analyte in the same day.

$\bar{X}$^b^: Mean recovery of triple analyses of the analyte in three consecutive days.

#### 3.2.5. Specificity

The proposed methods possessed high specificity according to ICH guidelines [33], as matrix component did not interfere with the absorption characteristics of the studied drugs; some of these excipients are magnesium stearate and dextrose in BTX and lactose monohydrate, microcrystalline cellulose, starch, povidone and magnesium stearate in LER. Furthermore, difference spectrophotometry method provides extra advantages in specificity as it is capable to remove any interference that could be gathered from absorbing co-formulated excipient.

### 3.3. Application

The developed methods were adopted for estimation of BTX alone and with co-administered LER in their laboratory-prepared mixtures and laboratory-prepared dosage forms, with ratio of 4 : 1 for BTX : LER, respectively. The analyses were carried out in triplicate at triple different concentrations of 5.0, 10.0, 15.0 µg ml^−1^ in method A, 30.0, 40.0, 50.0 µg ml^−1^ in method B and 8.0, 12.0, 16.0 µg ml^−1^ in method C for BTX. The concentrations of 2.0, 3.0, 4.0 µg ml^−1^ were used for analysis of LER in method C as abridged in table 3. The same procedures were performed as mentioned in Construction of calibration graphs, for each method. The low values of s.d. of the results and good agreement with other comparison ones when statistically analysed by *t*- and *F*-tests [34] reflected the applicability of these methods for quality control of dosage forms.

### 3.4. Greenness assessment

The main priority of the analyst is to achieve an eco-friendly method with high efficiency and low cost to be applied easily in pharmaceutical routine analysis. Green analytical chemistry aims to establish an ecological method that does not negatively affect the environment or deal with hazard chemicals. Greenness assessment of the proposed methods was achieved by using three analytical tools:

#### 3.4.1. Analytical eco-scale approach

Analytical eco-scale is one of the greenness assessments tools that is capable to evaluate the method efficiency and extract quantitative data about the compatibility of the method with the environment, taking into consideration the used reagents, instrument and the produced waste. This tool depends on calculating the analytical eco-scale value, which is obtained by allotting penalty points to all factors that negatively affect the environment and then subtracting the summation of all these points from basis of 100 [35]. The higher the eco-scale value obtained (more than 85), the excellent green profile of the method. The score higher than 50 reflects acceptable green analysis, while score lower than 50 reflects inadequately green analysis. Upon using this approach to assess the greenness of the proposed methods versus the comparison ones, we have found that method B was the greenest one, as the eco-scale value was 93 as shown in table 5. The eco-scale values of methods A and C were 85, which reflected also good green profile but less than method B. On the other hand, the reported HPLC methods [5,7] were less ecological because of using high amount of hazardous reagents, but they were still in the acceptable green analysis range with eco-scale values of 66 and 82, respectively.

**Table 5.** Analytical comparison of the greenness profile of the proposed spectrophotometric methods and the reported methods for the assay of the studied analytes by analytical eco-scale, GAPI and AGREE approaches.

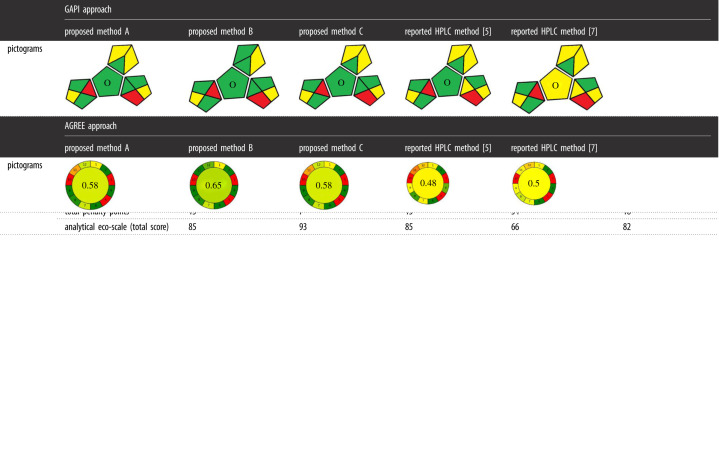

#### 3.4.2. Green analytical procedure index approach

Green analytical procedure index (GAPI) is a novel tool to evaluate the greenness of total methodology in the analytical procedure depending on 15 aspects. Five pentagrams, each one subdivided into three or four sections, were drawn and coloured according to the degree of environmental effect. Where green colour reflected low environmental impact, yellow colour expressed medium environmental impact and red colour ensured high environmental effect [36]. The proposed methods A and C showed six green shaded sections, while proposed method B exhibited seven green shaded sections. By contrast, only five and four green shaded sections were appeared in the compared HPLC methods [5,7], respectively, as shown in table 5. All these signs ensured the greenness of the proposed methods, especially method B, and reinforced its applicability in pharmaceutical routine analysis.

#### 3.4.3 Analytical GREEnness approach

Analytical GREEnness (AGREE) is the third approach employed in this study to evaluate the method greenness. This approach is recently developed by Pena-Pereira *et al*. [37] as a new tool for precise evaluation of the greenness of analytical methods. Greenness evaluation by this approach is based on measurement of the whole 12 SIGNIFICANCE parameters. The 12 principles appeared as segments; the colour of each segment is related to the green character. Each parameter has a score of 0.0–1.0 which is indicated by a specific colour in the pictogram (ranging from dark green (1.0) to red (0.0)). The final score is obtained by taking the average of the individual scores of the 12 parameters to give a score range of 0.0–1.0 where the ideal green methods have a maximum score of 1.0 [38]. Application of this approach contributes to evaluate the greenness of the proposed methods. The proposed method B was the greenest method which showed the highest score (0.65), followed by methods A and C which have the same score (0.58). On the other hand, the comparison methods showed loweer scores (0.50 and 0.48). These results ensured the greenness of the three proposed methods based on 12 principles of green analytical chemistry as shown in table 5.

## 4. Conclusion

Three different validated green spectrophotometric methods were performed for quantitative determination of the newly approved anti-coagulant drug, BTX. The three methods (zero-order spectrophotometry, difference spectrophotometry and first-order derivative spectrophotometry) were applied to assay BTX either alone or in combination with LER. The proposed methods were characterized by their simplicity, rapidity and ability to analyse multi-component mixture and to remove effects gathered from matrix background, degradation products or co-formulated absorbing excipients, as well as using of cheap and eco-friendly non-critical analytical reagent without the need for prior extraction or samples treatment. So, the proposed study was more consistent than the other reported studies and strongly recommends the application in quality control laboratories.

## Data accessibility

Data are available from the Dryad Digital Repository: https://doi.org/10.5061/dryad.931zcrjks [39].
